# Process Intensification of Chemical Exchange Method for Boron Isotope Separation Using Micro-Channel Distillation Technology

**DOI:** 10.3390/mi12101222

**Published:** 2021-10-06

**Authors:** Yin Tang, Yongjie Zheng, Jingzhi Tian, Jing Sun

**Affiliations:** 1 College of Light Industry and Textile, Qiqihar University, Qiqihar 161006, China; tangyin0001@163.com (Y.T.); zyj1964@163.com (Y.Z.); 2 College of Chemistry and Chemical Engineering, Qiqihar University, Qiqihar 161006, China; tjz6666@163.com; 3 Department of Academic Theory Research, Qiqihar University, Qiqihar 161006, China

**Keywords:** process intensification, boron isotope, micro-channel, distillation, 3D printing

## Abstract

A micro-channel distillation device was used for the process intensification method to separate boron isotopes, ^10^B and ^11^B. Three-dimensional (3D) printing technology was introduced to manufacture the micro-channel device, which used the chemical exchange method with anisole as the donor to separate the boron isotopes. This device was tested in total reflux mode, and the height of an equivalent theoretical plate of the micro-channel distillation equipment was reduced to 1.56 cm. The accurate control of pressure and temperature, as well as the flow rate of the complex, were factors that affected separation ability. Thus, for process intensification, this micro-channel distillation device can be operated horizontally and connected in series into similar modules to effectively improve separation efficiency and reduce the size of the equipment.

## 1. Introduction

**Figure 1:**
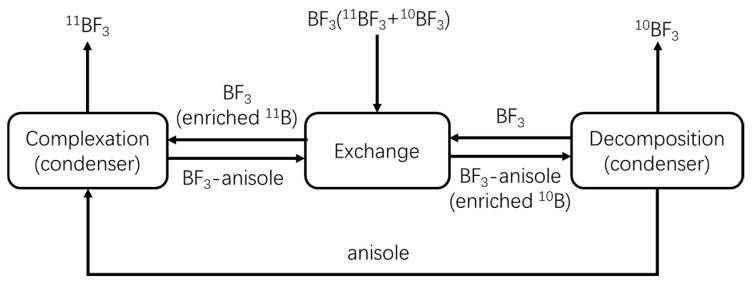
Principal scheme of the apparatus used for the separation of boron isotopes using the chemical exchange distillation method.

Due to their diverse nuclear properties, 10- and 11-boron isotopes are widely used in a variety of science and technology fields [1,2]. Methods for separating boron isotopes mainly include chemical exchange distillation [2,3], ion exchange resin [4,5,6], and laser-assisted retardation [7,8]. The chemical exchange distillation method with anisole as the donor has a high separation efficiency and can separate boron isotopes under normal temperatures and pressures, which makes it suitable for industrialization [9]. Chemical exchange distillation mechanisms, shown in Figure 1, generally include complexation, exchange, and decomposition.

The separation principle is based on the slight differences in the complexing ability of ^10^BF_3_ and ^11^BF_3_ with anisole, as expressed by Equation (1):

(1) B10F3(g)+B11F3⋅anisole(l)⇌αB11F3(g)+B10F3⋅anisole(l)

Isotope separation (or exchange) occurs when the gas phase is in contact with the BF_3_·anisole liquid complex, and the equilibrium constant of the two isotopes can be expressed in terms of α. During chemical exchange distillation, ^10^B and ^11^B can be separately isolated after several exchange equilibrium reactions. However, this process requires more than 100 theoretical plates and a fairly high column to complete the separation process [10].

Chemical process intensification technology was developed to address the high energy consumption, pollution, and low separation efficiencies in the chemical industry. Typical chemical process intensification technologies include high gravity [11], membrane process coupling [12,13], supercritical fluid [14], and microwave technology [15], which have been developed to reduce equipment size, simplify the process, reduce fuel consumption, and limit by-product generation. Thus, these microfabrication techniques allow for the fabrication of micro-structured devices [16], and techniques such as plasma-assisted ablation, chemical corrosion, and 3D printing are effective for chemical process intensification [17,18,19,20]. These microreactors, or micro-flow reactors, are devices that consist of single or multiple small-diameter channels, typically between 10 and 1000 μm [21,22]. In micro-fluidic environments, the operation principle is not applicable, as gravitational forces have less impact, while surface tension and viscosity dominate. Channel miniaturization can improve the reaction or separation rate and minimize side effects. In addition, the operating orientation of the fluid-like distillation process may become horizontal, as gravitational forces are insignificant compared to capillary forces, which are generated by surface tension. Bottenus et al. [23] tested a micro-channel distillation (MCD) device with this close-boiling system, starting with a feed of 1% propane in propylene, as a step toward considerably more difficult cryogenic separations of isotopes which could require upwards of 10,000 separation stages. Back et al. [24] reported an MCD column to reduce the overall size of distillation systems for ^136^Xe production. Both reducing the height of an equivalent theoretical plate (HETP) from the centimeter scale to the millimeter scale and operating in a more horizontal direction would make engineering a xenon isotope separation distillation system easier to engineer.

The basic principle of 3D printing technology is based on inkjet printing [25,26]. Three-dimensional printing fabricates a solid 3D structure, which is produced layer-by-layer through the deposition of suitable materials via an additive manufacturing machine [27,28,29]. This technology allows for the fabrication of complex mechanical devices, even enclosed and hollow structures at micro- or nano-scales [30]. In this study, a MCD chip was fabricated using 3D printing technology to separate ^10^B and ^11^B isotopes through chemical exchange distillation. A micro-channel with a small bulge may effectively limit the flow direction of liquids and horizontally perform the distillation operation. Thus, to investigate the separation effect of this device, micro-distillation in total reflux was performed.

## 2. Experimental Procedure

### 2.1. Reagents and Apparatus

Boron trifluoride gas was purchased from Zibo Linzi Xinqiang Chemical Co., Ltd., Zibo, China (CAS number: 7637-07-2, lot 20170905, purity ≥ 99.5%); anisole was purchased from Shanghai Aladdin Biochemical Technology Co., Ltd., Shanghai, China, (AR, purity ≥ 99.0%); and a 3–5 mm 4A molecular sieve was purchased from Shanghai Aladdin Biochemical Technology Co., Ltd., Shanghai, China. In addition, boric acid (catalog number: 951a, ^10^B/^11^B at 0.2473 ± 0.0002) was purchased from the National Institute of Standards and Technology (Gaithersburg, MD, USA), as a standard isotopic reference material.

A SimpNeed SLM 280 metal 3D printer system was manufactured by Hangzhou Niudai Tech Co., Ltd., Hangzhou, China. The impurities in anisole were measured using a GC-7820A gas chromatograph, manufactured by Agilent Technologies Inc, Santa Clara, USA. A muffle oven (SX2-12-10) was manufactured by Shanghai Xin Yi Instrument Co., Ltd. (Shanghai, China), and the residual boron in anisole was measured using a T6 UV–Vis spectrophotometer (Puxi Analytic Instrument Ltd., Beijing, China). The ^11^B/^10^B ratio was measured on an X7 series inductively coupled plasma mass spectrometer (ICP-MS) manufactured by Thermo Electron Corporation, Waltham, MA, USA. The high-precision intelligent temperature controllers (ST504-Q11) with a solid-state relay (SSR) and a corrosion-resistant PT100 thermal resistor were manufactured by Zhejiang Shangtong Instrument Co., Ltd., Leqing, China. The electric heating plates (20 × 50 mm, 20 W) were manufactured by Shenzhen Yiao electric heating appliance Co., Ltd., Shenzhen, China. The semiconductor chilling plates (TEC1-12706, 40 × 40 mm, 65 W) were manufactured by Jiangsu Xinghe Electronic Technology Co., Ltd., Suqian, China.

### 2.2. Dehydration and Purification of Anisole

Before creating the BF_3_-BF_3_·anisole system, the water in anisole was first removed, as the presence of water in the raw material causes a series of side reactions to occur; along with the generation of hydrogen fluoride and fluoroboric acid, anisole changes into phenol, which would destroy the complex equilibrium. Molecular sieve absorption was used, as it is a direct and effective method for removing water from organic solvents. The water content in anisole was reduced to less than 30 mg/L by adding an excessive amount of 4A molecular sieves into the flask. After dehydration, the dry anisole was transferred to a drying flask, and the distillate was collected to remove the solid particles in the solvent through distillation.

### 2.3. Manufacturing of the Micro-Distillation Device

**Figure 2:**
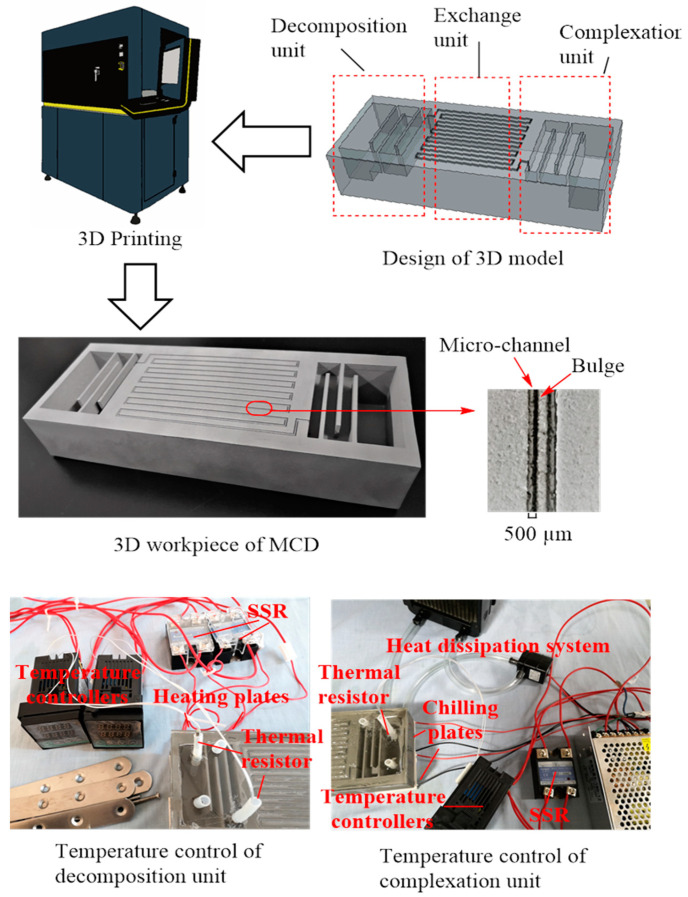
Design and manufacturing of micro-distillation workpiece based on 3D model by 3D printing and temperature control of the complexation unit and decomposition unit.

The main MCD workpiece was manufactured using a SimpNeed SLM 280 metal 3D printer system creating a software-designed 3D module, as shown in Figure 2. The material used for 3D printing the workpiece was 316L stainless steel. Due to the chromium-rich oxide film (passive film) on its surface, it has excellent corrosion resistance, and can withstand HF corrosion. Under the working conditions of this experiment, this material had good temperature resistance. The MCD device was composed of three sections: the complexation, exchange, and decomposition units.

#### 2.3.1. Complexation Unit

The complexation unit consisted of one large and three small, interconnected buffer cavities, in which the BF_3_ was complexed with anisole and produced heat, as shown in Equation (2):

(2) $$BF_{3}(g)+\mathrm{anisole}(l)\overset{cool}{\underset{heat}{⇌}}BF_{3}\cdot \mathrm{anisole}(l)$$

The small cavities were 10 mm in depth, while the large cavity was 30 mm in depth. The three smaller interconnected buffer cavities served to extend the residence and reaction times of the recombination reaction.

#### 2.3.2. Exchange Unit

The exchange unit was 10 cm in length, with a serpentine passageway that was 1.0 mm deep and 1.5 mm wide, comprising a series of interconnected micro-channels divided into two in the middle by a bulge 0.5 mm high and 0.5 mm wide. These channels were comprised of a set of gas–liquid micro-contactors, which acted as the main site of gas–liquid interphase mass transfer, and intraphase diffusion. Thus, ^10^B and ^11^B were concentrated in the liquid and gas phases, respectively.

#### 2.3.3. Decomposition Unit

The decomposition reaction was characterized as the reverse reaction of complexation (Equation (2)), with the decomposition unit used to separately decompose the BF_3_·anisole complex to BF_3_ (gas), and anisole (liquid) when heated. The structure of the decomposition unit was similar to the complexation unit, which also consisted of one large and three smaller buffer cavities. The depths of the smaller cavities in the decomposition unit were 20 mm and were deeper than those in the complexation unit. The complex in the complexation unit moved through the exchange unit and into the decomposition unit spontaneously, due to potential energy and capillary forces.

The temperature control of complexation unit and decomposition unit was realized by the combination of the thermal resistor, temperature controller, and solid-state relay. The connection mode of the sensor and the controller is shown in Figure 2.

### 2.4. Determination of ^11^B/^10^B Isotope Ratio

Before determination, the produced samples were first placed in a flask and then rinsed with ethanol. Distilled water was then added to the flask to dilute the samples to a boron concentration of approximately 1 mg/L. Then, ICP-MS was used to determine the ^11^B/^10^B isotope ratio. The operation method and experimental conditions of the ICP-MS experiments were similar to the methods described in Ref. [31]. After each test, solutions consisting of 2% of HNO_3_ and 0.1 mol/L of ammonia were alternately used to flush the system pipeline three times, to reduce error. The specific parameters used in the ICP-MS experiments for determining the boron isotope ratios (^11^B/^10^B) are shown in Table 1.

## 3. Results and Discussions

### 3.1. Production of Micro-Channel Distillation in Total Reflux

**Figure 3:**
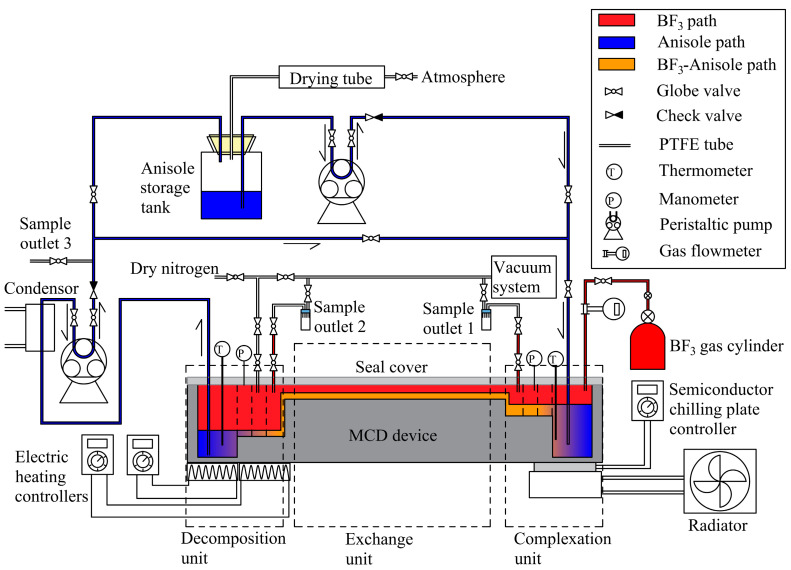
Process diagram of boron isotope separation by the micro-channel distillation device during operation.

The complete experimental process is shown in Figure 3. A pre-drilled quartz seal cover, metal straps, double-screw bolts, and silicone sealant were used to seal the upper portion of the device, while polytetrafluoroethylene (PTFE) tubes and valves were used as gas and liquid delivery lines, to prevent leakage due to corrosion. Two peristaltic pumps were installed along the liquid path as a power source for liquid transmission.

#### 3.1.1. Start-Up

The MCD device and its pipeline were pre-dried with nitrogen, and then the excess gas was repeatedly discharged using a vacuum. The anisole in the storage tank was pumped into the complexation unit by a peristaltic pump. Then, the semiconductor chilling plate and radiator were started to reduce the temperature in the complexation unit. When the BF_3_ gas cylinder valve was opened, BF_3_ entered the device and made contact with the anisole, and the complexing reaction proceeded in the complexation unit, releasing approximately 51.5 kJ/mol of heat [10]. The temperature of the complexation unit was maintained below 25 °C by adjusting the BF_3_ gas to a suitable flow rate.

**Figure 4:**
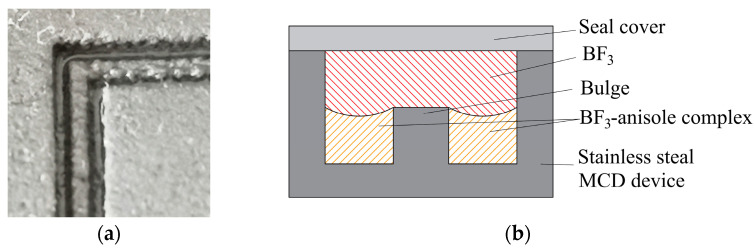
Gas–liquid distribution in the micro-channel with a bulge. (**a**) Wetted micro-channel; (**b**) gas–liquid distribution in micro-channel.

When the liquid level increased to a certain height, the BF_3_·anisole complex flowed continuously from the complexation unit to the exchange unit, under siphoning and capillary forces (Figure 4). Capillary forces prevented the liquid from overflowing into the gas path and causing flooding. When the complex filled the entire serpentine micro-channel, the complex flowed into the decomposition unit under the actions of potential energy and capillary action.

To promote the decomposition reaction, the temperature was raised to an appropriate value by adjusting the heating controller. This promoted the dissociation of BF_3_ from the liquid into the gas phase. Due to the low flow rate of the liquid, a trivial amount of energy was needed to raise the temperature. During complex decomposition, the concentration of anisole in the liquid phase increased and was continually enriched in the deep cavity of the decomposition unit. The anisole that decomposed from the complex was transported to the complexation unit through another peristaltic pump. Supplementation of raw materials, including the anisole and BF_3_, stopped when the pressure, liquid level, and temperature of the device reached equilibrium.

#### 3.1.2. Equilibrium Process

The MCD device was operated in total reflux. The temperature of the exchange unit was maintained at 25.0 ± 3.0 °C throughout the experiment. The BF_3_ was complexed with anisole, which subsequently reduced the pressure. The complex decomposed into BF_3_ gas, which elevated the pressure. The pressure difference between the complex and the decomposition units promoted the flow of BF_3_, and the countercurrent came into contact with the BF_3_·anisole complex in the serpentine micro-channel of the exchange unit. Mass transport occurred between the two phases along the flow length. Thus, ^10^BF_3_ was enriched in the decomposition unit, while ^11^BF_3_ was enriched in the complexation unit upon reaching equilibrium.

#### 3.1.3. Sample Collection and Testing

The enriched ^11^B and ^10^B samples were separately collected from outlets one and two after achieving equilibrium. The division of the two isotopes was characterized by changes in the ^11^B/^10^B ratio. The main component of the samples collected from outlet three was anisole, which was stripped from the BF_3_·anisole complex. Residual boron content was thus used to characterize the complex decomposition effect of the decomposition unit, which was determined by spectrophotometry. By-products, such as phenol, generated by the complex and decomposition reactions, were analyzed by gas chromatography.

To clearly show the experimental process, the workflow, procedure, and validation for the separation of boron isotopes by MCD is shown in Table 2.

### 3.2. Range of Flow Rate and Equilibrium Time

The flow rate of the complex was controlled by adjusting the flow rate of the pump. The following lists the three restrictions set for the flow rate:

1. The liquid level in the complexation unit could not pass through the serpentine micro-channel; otherwise, flooding would occur.
2. The feed volume of the decomposition unit could not exceed the load of the heater. Although the heating temperature of the heater could be increased to accelerate the decomposition of the complex, excessive local temperatures would increase the chance of side reactions.
3. The anisole in the decomposition unit could not be completely extracted; otherwise BF_3_ would overflow into the pump.

Therefore, for these reasons, the optimal flow rate of the pump was determined to be 0.18 mL/min, after comparison and testing. If the flow rate of the pump was increased to maximum, the liquid level difference between the complexation and the decomposition units would increase, thus increasing the potential energy differences of the liquid and the flow rate of the fluid in the micro-channel. This operation is similar to the increase in gas flow rate and reflux in traditional distillation, which is beneficial for improving mass transfer efficiency.

According to the isotopic fractionation factor of the BF_3_-BF_3_·anisole system, ^11^BF_3_ and ^10^BF_3_ were regarded as the light and heavy components, respectively. When the isotope ratio was constant, separation was considered to be in equilibrium. The distribution equilibrium of the two isotopes in MCD was based on the balance of the convection and diffusion of laminar flow in the micro-channel. The complexation, exchange, and decomposition units for the micro-distillation system were regarded as the condenser, packing layer, and reboiler in a traditional rectification device, respectively.

The ^11^B/^10^B isotope ratio of the BF_3_ raw material, as detected by ICP-MS, was 3.981 ± 0.005. Samples were collected from sample outlets one and two for five hours, which did not significantly affect system stability. The ^11^B/^10^B isotope ratio displayed steady growth with time.

**Figure 5:**
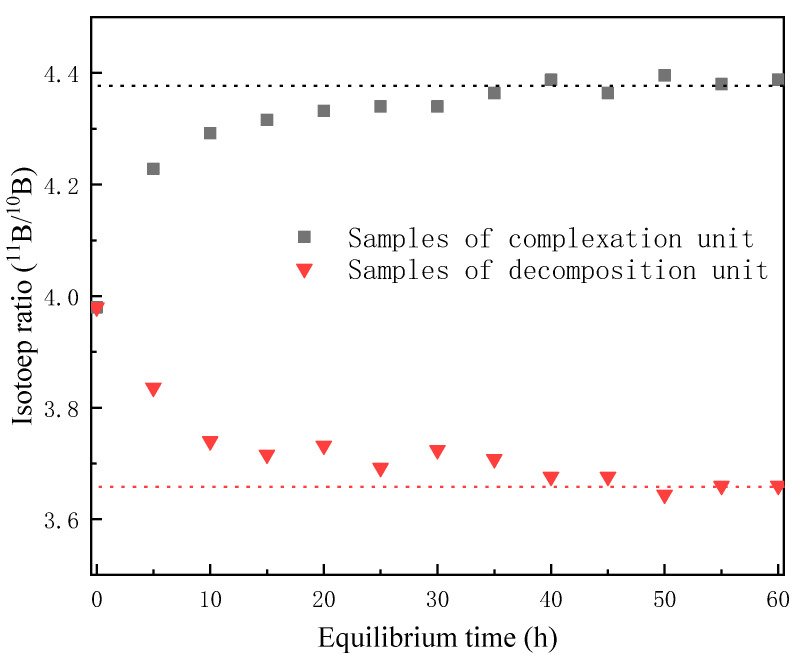
Stability of the ^11^B/^10^B isotope ratios in total reflux mode.

Under a flow rate of 0.15 mL/min and pressure of 5.0 kPa, the total separation increased rapidly with time from initial onset, and gradually plateaued after 40 h, as shown in Figure 5. The slight fluctuation, possibly caused by the fluctuation in temperature in the decomposition unit, did not significantly impact the trend. The ^11^B/^10^B isotope ratios of the samples stabilized around 4.38 and 3.66 for the complexation and decomposition units, respectively.

### 3.3. Testing of Height Equivalent to a Theoretical Plate

**Figure 6:**
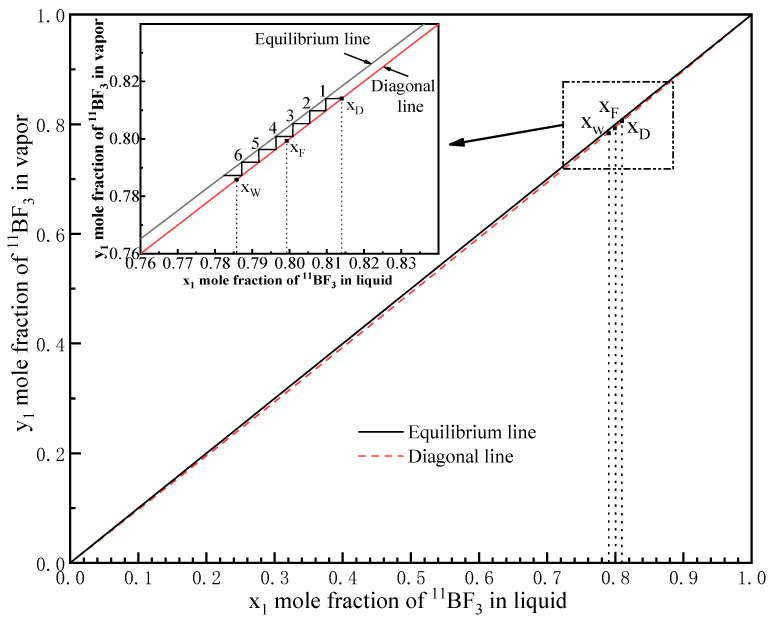
McCabe-Thiele method for evaluating the separation of ^11^BF_3_ and ^10^BF_3_ with total reflux.

The ^11^B/^10^B equilibrium line (curve) was flat and ran close to the diagonal line of the McCabe-Thiele diagram, which presents the separation effect, as shown in Figure 6. According to the *x_D_* and *x_W_* results, the horizontal and vertical lines were drawn between the equilibrium line and the diagonal line.

The MCD unit operated under total reflux; thus, the theoretical number of separation stages (*N_min_*) could be calculated from the mole fractions of the distillate (collected from the complexation unit) and the bottom (collected from the decomposition unit), using the Fenske equation:

(3) $$N_{min}=\frac{\log[(\frac{x_{D}}{{1-x}_{D}})(\frac{{1-x}_{W}}{x_{W}})]}{\log(a)}$$

where *x_D_* and *x_W_* are the mole fractions of ^11^BF_3_ in ^10^BF_3_ and ^11^BF_3_, in the complexation and decomposition units, respectively. The HETP was calculated by dividing the liquid length of the exchange unit in the MCD device, *L_MCD_*, by the number of theoretical stages, according to:

(4) $$\mathrm{HETP}=\frac{L_{\mathrm{MCD}}}{N_{\min}}$$

where *N_min_* was 6.28, as calculated by the Fenske equation. Hence, the HETP was consequently 1.59 cm for the length of the exchange unit of the MCD, which was 10 cm. Thus, MCD showed good separation efficiency according to these results, which could be optimized with further refinement.

### 3.4. Effect on Flow Rate and Pressure

Additional experiments were performed to observe the effects of different flow rates and pressure, as shown in Table 3. With an increase in flow rate, the separation efficiency improved. The minimum theoretical plate number increased slightly with increased pressure. The minimum HETP was as low as 1.56 cm under the flow rate conditions of 0.15 mL/min, and a pressure of 10.0 kPa. However, if pressure rose continuously, decreases in HETP were difficult. This was likely due to the collision probability between BF_3_ and anisole with slight increases in pressure. Meanwhile, the increase in pressure caused the complex ratio to increase, which heightened the surface level of the liquid phase inside of the micro-channel, and effectively intensified the mass transfer process. After each test, the phenol content in anisole was less than 0.1 mg/L, indicating that this method had no obvious effects on anisole modification.

### 3.5. Residual Boron Content in the Steam of the Decomposition Unit Steam

The increase in pressure intensified the temperature fluctuations in the decomposition unit, which increased the decomposition temperature as well as the heat load decomposition. This resulted in an increase in the amount of ^10^B-enriched boron–fluoride that remained in the anisole. In addition, the ^11^B/^10^B ratio of the complexation unit decreased, because the 10B-enriched boron–fluoride was transported by the peristaltic pump.

**Figure 7:**
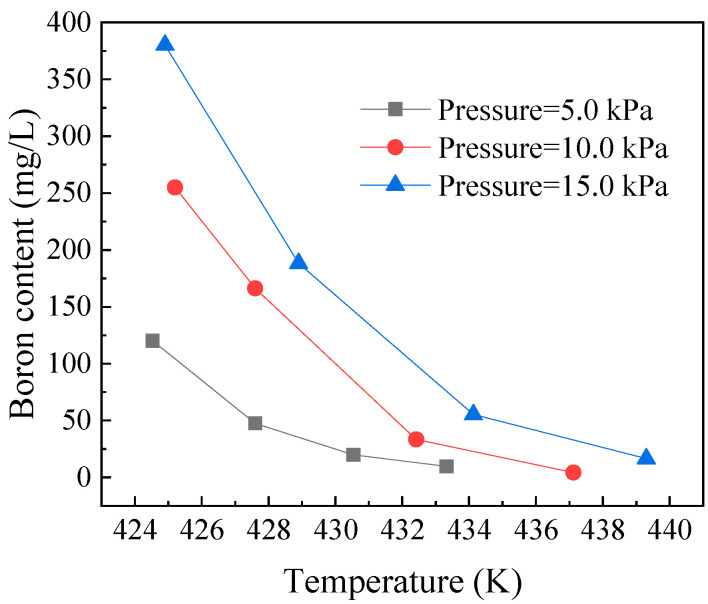
Residual boron content in anisole withdrawn from the decomposition unit under various pressures.

To investigate the effects of decomposition temperature on residual boron content, a series of experiments were carried out at a flow rate of 0.15 mL/min, as shown in Figure 7. Even under different pressure conditions, boron content could be reduced to below the ideal value by properly increasing the decomposition temperature. However, excessively high temperatures accelerated the decomposition of anisole and increased system pressure fluctuations. Thus, boron trifluoride may form complexes with some by-products, such as phenol, and these by-products do not easily decompose. Thus, if the operation time is prolonged continuously, the boron content in anisole would continue to accumulate. Hence, an additional purification device could be used to remove these excess by-products, to avoid excessive boron transport into the complexation unit.

## 4. Conclusions

The separation of boron isotopes by chemical exchange distillation was assessed using a micro-channel distillation device for the process intensification method in total reflux mode. Three-dimensional printing technology was utilized to manufacture the main body of the MCD device, which simplified the production of the device, allowing it to be industrially produced. Under optimized experimental conditions, the HETP of the equipment reached 1.56 cm. Heat transfer and pressure were precisely controlled, as these factors affected the separation effect. The decomposition amounts of anisole and boron residue could be controlled within an acceptable range by adjusting the parameters, thus validating this method. Moreover, due to capillary action, the effect of gravity was reduced, and the operation direction of the equipment was modified from the traditional transverse to the longitudinal direction.

The height of traditional distillation columns may be a challenge for engineers, but it is not necessarily impossible. One technology that may make engineering easier is MCD. As a potential technology, MCD is expected to be combined with a variety of emerging manufacturing methods to show its unique advantages in the future.

## Figures and Tables

**Table 1:** Experimental conditions and ICP-MS parameters.

Item	Parameter	Item	Parameter
Nebulizer	Microconcentric nebulizer	Sensitivity (s^−1^(μg·L^−1^) ^−1^)	1.759 × 10^6^ CPS
Spray chamber temperature (°C)	3	Scanning mode	Peak-jump
Nebulizer gas flow (L·min^−1^)	0.93	Dwell time (ms)	10
Auxiliary gas flow (L·min^−1^)	0.9	Acquisition degree	10
Cool gas flow (L·min^−1^)	10	Acquisition time (s)	20
Plasma power (W)	1250	Channels per AMU	3
Resolution	Standard	Runs (replicates)	3
Sample uptake rate (mL·min^−1^)	1	Sample depth (mm)	104
Ionization lens parameters	L1 3.8; L2 31.8; L3 189.8	-	-

**Table 2:** Workflow, procedure, and validation for the separation of boron isotopes by MCD.

Workflow	Procedure	Validation
Start-up	Pre-dry	Until the device is completely dry
	Supplementation of anisole	-
	Start chilling plate and radiator	Temperature control range of decomposition unit was 155–160 °C; Temperature control range of complexation unit was 5–25 °C
	Supplementation of BF_3_	-
	Adjusting the BF_3_ gas to a suitable flow rate	Keep the temperature of complexation unit below 25 °C
	Circulation of liquid	Transport anisole from decomposition unit to the complexation unit
	Supplementation Stop	Pressure, liquid level, and temperature of the device reached equilibrium
Equilibrium process	Maintain stability	Keep the pressure, liquid level, and temperature stable
	Maintain the temperature of the exchange unit	Keep the exchange unit near 25.0 ± 3.0 °C
Sample collection and testing	Sample from outlets 1 and 2	Investigate the abundance of ^11^B and ^10^B
	Sample from outlets 3	Testing residual boron content and by-products

**Table 3:** Effects of pressure on stability time and HETP.

Run	Flow Rate (mL/min)	Pressure (kPa) ^1^	Stable Time (h)	*N_min_*	HETP (cm)	Phenol Content (mg/L) ^2^
1	0.15	1.0	40	6.10	1.64	0.057
2	0.15	5.0	40	6.28	1.59	0.042
3	0.15	10.0	40	6.43	1.56	0.043
4	0.15	15.0	45	6.41	1.56	0.092
5	0.08	5.0	40	5.25	1.90	0.051
6	0.08	10.0	45	5.44	1.84	0.067
7	0.04	5.0	45	2.98	3.36	0.086
8	0.04	10.0	45	3.20	3.12	0.058

^1^ Pressure data were obtained by measuring the pressure of the complexation unit. The fluctuation range of the pressure was within 0.2 kPa. ^2^ Samples of phenol content in anisole were collected from sample outlet 3 and determined after continuous operation for 60 h.

## Data Availability

Data available in a publicly accessible repository.

## Abbreviations

The following abbreviations are used in this manuscript:

MCD	Micro-channel distillation
HETP	Height of an equivalent theoretical plate
ICP-MS	Inductively coupled plasma mass spectrometry
CPS	Count per Second
PTFE	Polytetrafluoroethylene
SSR	Solid-state relay
